# INNOVATIVE STRATEGIES TO SUPPORT OLDER ADULTS AND THEIR FAMILIES IN HOSPICE AND PALLIATIVE CARE

**DOI:** 10.1093/geroni/igz038.718

**Published:** 2019-11-08

**Authors:** George Demiris, Karen Hirschman

**Affiliations:** University of Pennsylvania, Philadelphia, Pennsylvania, United States

## Abstract

In order to better support older adults with life-limiting illness and their families, many initiatives utilize information technology and other innovative platforms to increase access to supportive services and bridge geographic distance. Such technologies cover a broad range of systems ranging from smart phone applications to wearables and traditional telehealth platforms. There is a growing evidence base for such interventions but technical, clinical and ethical challenges remain when utilizing technology in the context of hospice and palliative care especially for older adults, including the concerns for caregiver burden, privacy, security, confidentiality, obtrusiveness and accessibility. In this symposium we provide an overview of innovative tools available for interventions in palliative and hospice care designed for patients and/or family caregivers in urban and rural settings. We provide lessons learned from three NIH funded studies testing different technology-based interventions in various settings including home hospice and outpatient palliative care. Discussion will follow focused on the clinical, ethical and practical challenges of innovation and the unique considerations for technology-mediated intervention design in a variety of palliative and hospice care settings. This symposium aims to provide: 1. an overview of existing technology-based interventions for older adults and their families in palliative care and hospice 2. evidence-based recommendations resulting from clinical trials in urban and rural settings for the design and implementation of innovative tools in hospice and palliative care 3. a discussion of challenges and opportunities for the use of technology to support older adults and their families

